# Monthly dynamic associations between cost-of-living pressure and depressive and anxiety symptoms: a longitudinal study based on cross-lagged network analysis

**DOI:** 10.3389/fpsyt.2026.1914441

**Published:** 2026-08-19

**Authors:** Zhang Lin, Xiong-Zhe Gai, Hui-Hui Yao

**Affiliations:** 1 Department of Clinical Psychology, Wenzhou Seventh People's Hospital, Wenzhou, China; 2 Wenzhou Medical University, Wenzhou, China

**Keywords:** anxiety, CLPN, cost-of-living pressure, cross-lagged panel network, depression, GAD-7, PHQ-8

## Abstract

**Background:**

Cost-of-living pressure is an important social determinant of public mental health, but its short-term associations with specific depressive and anxiety symptoms remain unclear.

**Methods:**

We analyzed 12 monthly waves from the Changing Cost of Living Study. A pooled lag-1 cross-lagged panel network was estimated to examine group-level temporal predictive associations among five economic-pressure indicators, eight PHQ-8 depressive symptoms, and seven GAD-7 anxiety symptoms. The analysis included 4,085 complete lag-1 observations from 471 participants, adjusting for country, age, gender, subjective socioeconomic status, and wave. Robustness checks used a conservative lambda.1se network, deletion sensitivity analyses for overlapping symptom items, and bootstrap stability assessment.

**Results:**

Economic-pressure nodes showed high autoregressive stability, particularly housing risk, employment risk, food insecurity, poverty/deprivation risk, and difficulty managing finances. Within-symptom paths were stronger than cross-domain paths. Stable bidirectional associations emerged between anhedonia and depressed mood, uncontrollable worry and excessive worry, and psychomotor problems and restlessness. Some economic pressure-to-symptom paths were detected in the lambda.min model, but these small paths were not retained in the more conservative lambda.1se model.

**Conclusions:**

At the group level, monthly temporal predictive patterns under cost-of-living pressure were characterized mainly by persistence within economic pressures and reciprocal associations within depressive and anxiety symptoms. Cross-domain economic pressure-symptom associations were weak and should not be interpreted as causal effects or as evidence of a uniform individual-level process.

## 1 Introduction

In recent years, cost-of-living pressure has become an important macro-level factor affecting public mental health. Rising energy, food, housing, and employment-related pressures expose many households to multidimensional economic strain, including difficulty managing finances, food insecurity, housing risk, and employment risk. Beyond material deprivation, these pressures may influence depressive and anxiety symptoms through chronic stress, threat anticipation, resource depletion, and constrained behavioral choices (1, 2). Although economic hardship is a well-established social determinant of depression and anxiety, cost-of-living pressure is often more short-term, fluctuating, and multidimensional, requiring higher-resolution analysis of its dynamic associations with specific symptoms.

Previous research has established a strong link between economic pressure and mental health. Systematic reviews associate financial hardship with depression, anxiety, and psychological distress, through mechanisms such as shame, loss of control, rumination, future uncertainty, reduced social participation, and diminished coping resources (3, 4). Prior research suggests bidirectional associations between poverty and mental health: poverty has been associated with stress, physiological load, and reduced cognitive bandwidth, while depression and anxiety have been associated with reduced work capacity, decision-making, and social functioning (2). However, longitudinal studies vary in measurement frequency, economic-pressure dimensions, symptom granularity, and directionality estimation (5). In the cost-of-living crisis context, the Changing Cost of Living Study showed that monthly financial changes among adults in the United Kingdom and France were closely associated with changes in anxiety and depression (6), and that food insecurity had concurrent and within-person associations with PHQ-8 and GAD-7 symptoms (7). Broader evidence also links food insecurity and housing insecurity to poorer mental health (8, 9). Thus, cost-of-living pressure should be treated not as a single income variable, but as an interconnected system involving food insecurity, poverty/deprivation risk, housing risk, employment risk, and difficulty managing finances.

Recent studies further contextualize this topic while using different populations and designs. In a two-wave Canadian study of older adults, perceived increases in cost of living were associated with higher psychological distress at follow-up (10). A Bolivian survey conducted during COVID-19 described heterogeneity in economic well-being and self-reported anxiety and depression across sociodemographic groups (11). In a large Norwegian adult sample, cross-sectional network analysis linked multiple financial factors with individual depressive and anxiety symptoms (12). These complementary studies motivate symptom-specific investigation, but they differ from the present monthly pooled CLPN in setting, population, measurement schedule, and analytic design.

Drawing on the stress-process model and conservation-of-resources theory, cost-of-living pressures can be conceptualized as distinct but related stress exposures. Housing risk may heighten threat anticipation and perceived loss of control; employment risk may increase uncertainty regarding future resources; and food insecurity or difficulty managing finances may constrain access to basic resources and reduce coping capacity. These perspectives complement financial-strain research by specifying plausible psychosocial pathways. Although the present pooled CLPN does not test these mechanisms directly, they provide a theory-driven rationale for examining whether specific pressures show distinct temporal predictive associations with particular symptoms (3, 4, 13, 14).

Although existing research clearly demonstrates associations between economic pressure and depression and anxiety, three key gaps remain in the literature. First, many studies use total scale scores or a single indicator of economic hardship, making it difficult to identify which specific economic-pressure nodes have temporal predictive associations with which specific depressive or anxiety symptoms, even though data-driven item-level analyses have shown that individual depression items carry distinct predictive information (15). Second, most studies use cross-sectional designs or longitudinal designs with long time intervals, which are poorly suited to capturing short-term dynamics of cost-of-living pressure at a monthly scale; longitudinal studies also indicate marked heterogeneity in depressive symptom trajectories across individuals (16). Third, existing research often assumes that economic pressure is an upstream risk factor for psychological symptoms, but less often simultaneously tests whether symptoms predict subsequent economic pressure. As a result, it remains difficult to distinguish among economic pressure predicting symptoms, symptoms predicting later economic pressure, and the possibility of limited coupling between the two.

Cross-lagged network analysis provides a useful framework for examining how cost-of-living pressure and depressive-anxiety symptoms influence one another over time. Network theory views mental disorders as dynamic interactions among symptoms, and prior studies show that clinically meaningful depression-anxiety associations often occur at the symptom level rather than only at the diagnostic-total level (17–20). Compared with traditional variable-level models, the cross-lagged panel network (CLPN) estimates directional temporal predictive paths among nodes while adjusting for other nodes, helping identify convergence points and predictive sources in later network states (21). Although CLPNs have been applied to depressive and anxiety symptoms (22, 23), no study has yet integrated multiple cost-of-living pressure nodes with PHQ-8 and GAD-7 symptoms in a monthly cross-lagged network. To address this gap, the present study used 12 monthly waves from the Changing Cost of Living Study to construct a pooled lag-1 CLPN including PHQ-8 symptoms, GAD-7 symptoms, and five economic-pressure nodes (FI, DEST, HOUSE, EMPLOY, and FINDIFF). We examined monthly predictive patterns, key within- and cross-domain paths, and node-level indices including In-Expected Influence, Out-Expected Influence, and predictability, expecting economic pressure and symptoms to form a multidimensional, short-term, partly bidirectional system rather than a strongly one-way process.

## 2 Materials and methods

### 2.1 Study design and data source

This study was a secondary analysis of publicly available longitudinal data from the Changing Cost of Living Study. The original study conducted monthly online surveys in the United Kingdom and France to track participants’ economic circumstances, health status, depressive and anxiety symptoms, and related psychosocial variables during a period of rising living costs. The present study used 12 consecutive monthly waves from September 2022 to August 2023 and organized adjacent monthly records into 11 lag-1 transitions to estimate monthly cross-lagged networks.

### 2.2 Network nodes

The main network comprised 20 z-standardized nodes: eight PHQ-8 depressive symptoms, seven GAD-7 anxiety symptoms, and five economic-pressure indicators. PHQ-8 and GAD-7 items were recoded from 1–4 to 0-3, with higher scores indicating greater symptom severity. Economic-pressure nodes included food insecurity (FI, 0-3), poverty/deprivation risk (DEST, 0-100), housing risk (HOUSE, 0-100), employment risk (EMPLOY, 0-100), and difficulty managing finances (FINDIFF, reverse-coded, 1-5), all coded so higher values reflected greater economic pressure. All nodes were z-standardized using the full longitudinal sample before model entry. Subjective socioeconomic status was assessed on a 1–10 ladder, with higher values representing a higher perceived socioeconomic position.

### 2.3 Covariates and data preprocessing

For each participant, data from two adjacent months were paired as records at month t and month t + 1. The 20 network nodes at month t were used to predict each network node at month t + 1. Country, age, gender, subjective socioeconomic status, and wave were included as covariates. Country, gender, and wave entered the model as categorical variables, whereas age and subjective socioeconomic status entered as continuous variables. The main analysis used a complete-case strategy, requiring complete data on all network nodes and covariates at both month t and month t + 1; missing values were not imputed. The complete-case requirement provided a common observed-data matrix across all nodewise regressions and lag-1 transitions and avoided introducing imputed values into the network estimation. Because the same participant could contribute multiple adjacent-month lag-1 records, this study estimated a pooled lag-1 group-average temporal predictive network. Accordingly, estimated paths represent conditional group-level temporal predictive associations in the pooled analytic sample; they do not estimate person-specific change processes, establish causality, or imply homogeneous associations across individuals. Educational attainment and citizenship/immigration status were not available in the public analytic dataset and therefore could not be included as covariates.

### 2.4 Estimation of the cross-lagged network

We estimated the pooled lag-1 CLPN using nodewise LASSO-regularized Gaussian regression. For each month t + 1 node, a separate regression was fitted with all 20 month t nodes and covariates as predictors. Network-node coefficients were LASSO-penalized, while covariates (country, age, gender, subjective socioeconomic status, and wave) were left unpenalized to ensure consistent adjustment.

The penalty parameter was selected by 10-fold cross-validation, with the main model using the lambda.min rule. In the adjacency matrix, rows represented month t predictors and columns represented month t + 1 outcomes; diagonal entries indicated autoregressive paths and off-diagonal entries cross-lagged paths. Because all nodes were z-standardized, LASSO-selected edges are standardized conditional coefficients rather than significance-test results. Outputs included nonzero edges, edge-weight rankings, node predictability (R²), and In-/Out-Expected Influence, with centrality interpreted alongside bootstrap stability results.

### 2.5 Robustness and sensitivity analyses

Robustness analyses included three steps. First, the 20-node network was re-estimated using the more conservative lambda.1se rule to determine which main-model patterns remained under stricter regularization. Second, because PHQ8 psychomotor problems and GAD5 restlessness may overlap in item content, symptom-subnetwork deletion analyses removed PHQ8, GAD5, or both and compared the resulting networks using edge-weight correlations and signed Jaccard similarity. Third, nonparametric and case-dropping bootstrap analyses were performed for the 20-node main network (nBoots = 500) to assess edge-weight uncertainty and the stability of edge weights, In-Expected Influence, and Out-Expected Influence. Because participants could contribute multiple lag-1 records, bootstrap results were interpreted as internal stability evidence for the pooled network.

### 2.6 Network visualization and statistical software

Autoregressive paths were omitted from network plots, and major stable paths were shown in the main figures to improve directional clarity and readability. Full edge-weight rankings, weak economic pressure-symptom paths, centrality, and predictability results are reported in tables and **Supplementary Materials**. Positive and negative edges were distinguished by different colors in visualizations, and node colors were used to distinguish depressive symptoms, anxiety symptoms, and economic-pressure nodes.

Data cleaning and descriptive statistics were performed in Python. CLPN estimation, centrality computation, bootstrap stability analyses, and network visualization were performed in R 4.5.0, using packages including glmnet, qgraph, bootnet, dplyr, and ggplot2. All analyses were conducted using scripts to ensure reproducibility.

## 3 Results

### 3.1 Analytical sample and descriptive characteristics

The final economic pressure-extended main model included 4,085 complete lag-1 observations from 471 participants across 11 adjacent monthly transitions. The mean age of participants was 41.89 years (SD = 10.52), and the mean subjective socioeconomic status was 5.62 (SD = 1.84). The sample included 242 participants from the United Kingdom (51.4%) and 229 from France (48.6%); 237 participants were women (50.3%), 231 were men (49.0%), and 3 reported PNTS or self-described gender (0.6%).

In the main-model sample, the mean PHQ-8 total score was 6.02 (SD = 5.61, median = 5), and the mean GAD-7 total score was 5.51 (SD = 5.39, median = 4). Among economic-pressure nodes, the mean score was 0.29 (SD = 0.77) for food insecurity (FI), 23.96 (SD = 25.93) for poverty/deprivation risk (DEST), 21.91 (SD = 25.53) for housing risk (HOUSE), 30.67 (SD = 30.12) for employment risk (EMPLOY), and 2.43 (SD = 1.03) for difficulty managing finances (FINDIFF). These descriptive results indicate that the sample, on average, showed mild to moderate depressive and anxiety symptom levels, with substantial individual differences in economic-pressure indicators.

Across the 12 scheduled waves, records were available for 472, 443, 422, 411, 417, 419, 398, 400, 392, 375, 361, and 372 participants, respectively, among the 485 participants with at least one study record. Of these participants, 471 contributed at least one complete lag-1 record and 14 contributed none. At the first available assessment, the two groups showed no statistically detectable differences in age, subjective socioeconomic status, PHQ-8, or GAD-7 (permutation p values = .53-1.00), but their country (France: 78.6% vs. 48.6%, p = .031) and gender distributions differed (p <.001); estimates for the 14 noncontributors require caution.

### 3.2 Main economic pressure-symptom CLPN

**Figure 1:**
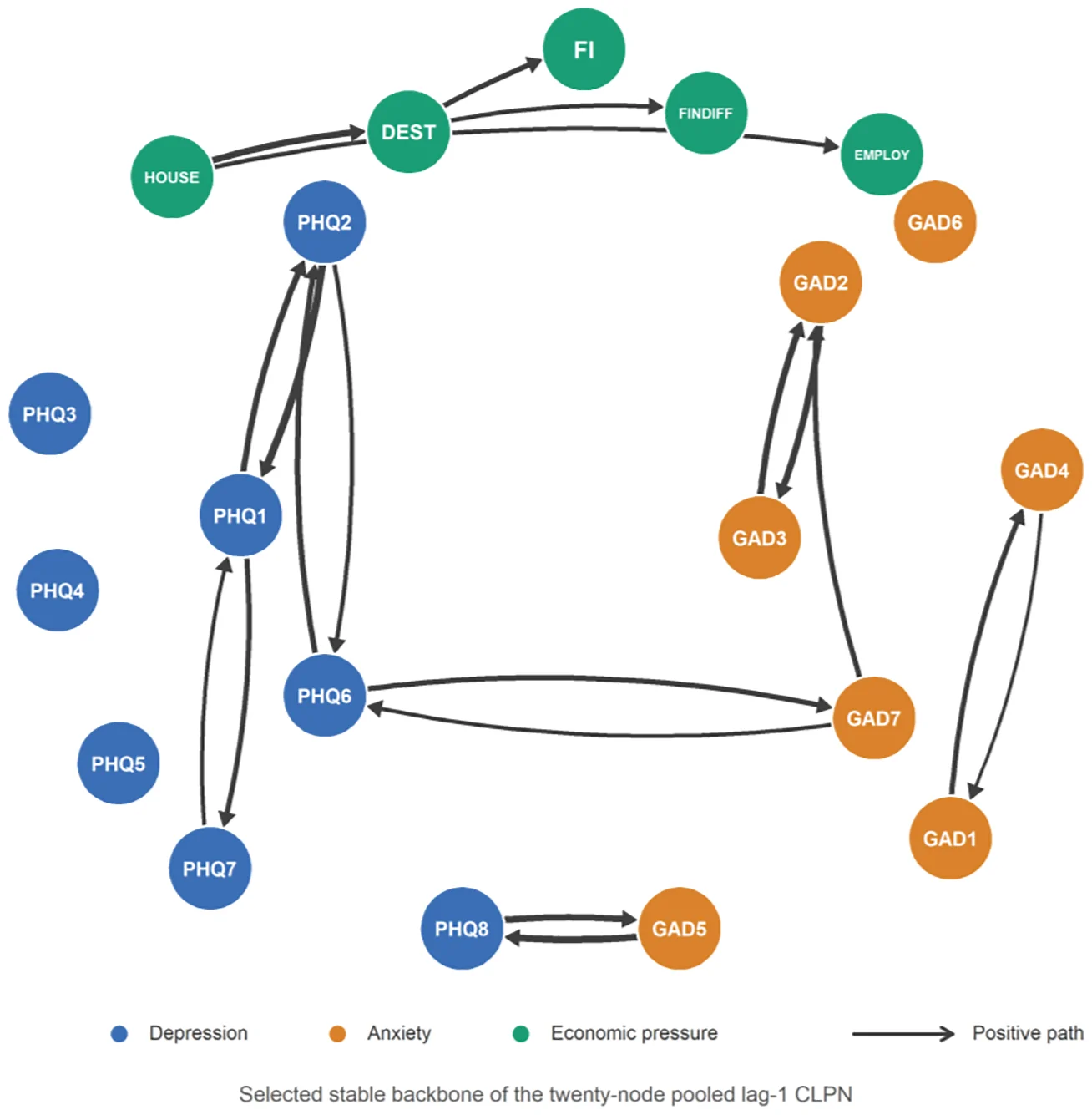
Selected stable backbone network of the 20-node pooled lag-1 CLPN. Autoregressive paths were omitted, and only major stable paths are shown to improve directional clarity and visual readability. All paths shown are positive predictive paths; weak economic pressure-symptom paths are presented separately in the key-path figure. Green nodes represent economic-pressure nodes, blue nodes represent PHQ-8 depressive symptoms, and orange nodes represent GAD-7 anxiety symptoms. FI, food insecurity; DEST, poverty/deprivation risk; HOUSE, housing risk; EMPLOY, employment risk; FINDIFF, difficulty managing finances.

After controlling for country, age, gender, subjective socioeconomic status, and wave, the 20-node pooled lag-1 CLPN yielded 288 nonzero edges, including 20 autoregressive edges and 268 nonautoregressive cross-lagged edges, with a mean node predictability of R2 = .614. Among the 268 nonautoregressive edges, 249 were positive and 19 were negative, indicating that nonautoregressive paths in the main network were predominantly positive temporal predictions. Negative paths were generally small in weight. The larger negative edges included GAD3 -> PHQ7 (-0.052), GAD2 -> PHQ5 (-0.039), EMPLOY -> FI (-0.036), and DEST -> PHQ7 (-0.034). Therefore, the present article focuses primarily on positive paths with larger weights and greater stability. The selected stable backbone of the 20-node pooled lag-1 CLPN is shown in **Figure 1**.

All 20 nodes had nonzero autoregressive edges. Economic-pressure nodes showed overall higher autoregressive stability than symptom nodes. Higher autoregressive paths included HOUSE -> HOUSE (0.727), EMPLOY -> EMPLOY (0.720), FI -> FI (0.670), FINDIFF -> FINDIFF (0.629), and DEST -> DEST (0.561). Among symptom nodes, higher autoregressive paths included PHQ6 -> PHQ6 (0.540), PHQ3 -> PHQ3 (0.512), and PHQ5 -> PHQ5 (0.490). **Figure 1** presents a stable backbone selected from the main network to enhance readability, rather than the complete set of 268 nonautoregressive edges; the full edge-weight ranking should be interpreted together with the tables in the main text and **Supplementary Materials**.

### 3.3 Economic-pressure subnetwork

Economic-pressure nodes formed a relatively clear positive temporal predictive subnetwork. The strongest within-economic path was HOUSE -> DEST (0.140), suggesting that higher housing risk predicted greater poverty/deprivation risk in the following month. Other relatively strong paths included DEST -> FI (0.108), HOUSE -> EMPLOY (0.084), DEST -> FINDIFF (0.084), EMPLOY -> HOUSE (0.065), and FINDIFF -> FI (0.063). These results indicate clear monthly persistence and mutual prediction among economic-pressure indicators, especially among housing risk, poverty/deprivation risk, food insecurity, and difficulty managing finances.

### 3.4 Temporal predictive paths between economic pressure and symptoms

Several cross-domain temporal predictive paths were observed between economic pressure and symptoms, but their weights were generally small. In the direction from economic pressure to subsequent symptoms, larger paths included HOUSE -> PHQ7 (0.053), HOUSE -> GAD7 (0.051), EMPLOY -> GAD1 (0.045), FI -> PHQ5 (0.041), EMPLOY -> PHQ5 (0.041), and FI -> GAD5 (0.040). These results suggest that housing risk, employment risk, and food insecurity may have weak monthly associations with subsequent concentration difficulties, fear that something awful might happen, nervousness/anxiety, appetite problems, and restlessness.

**Figure 2:**
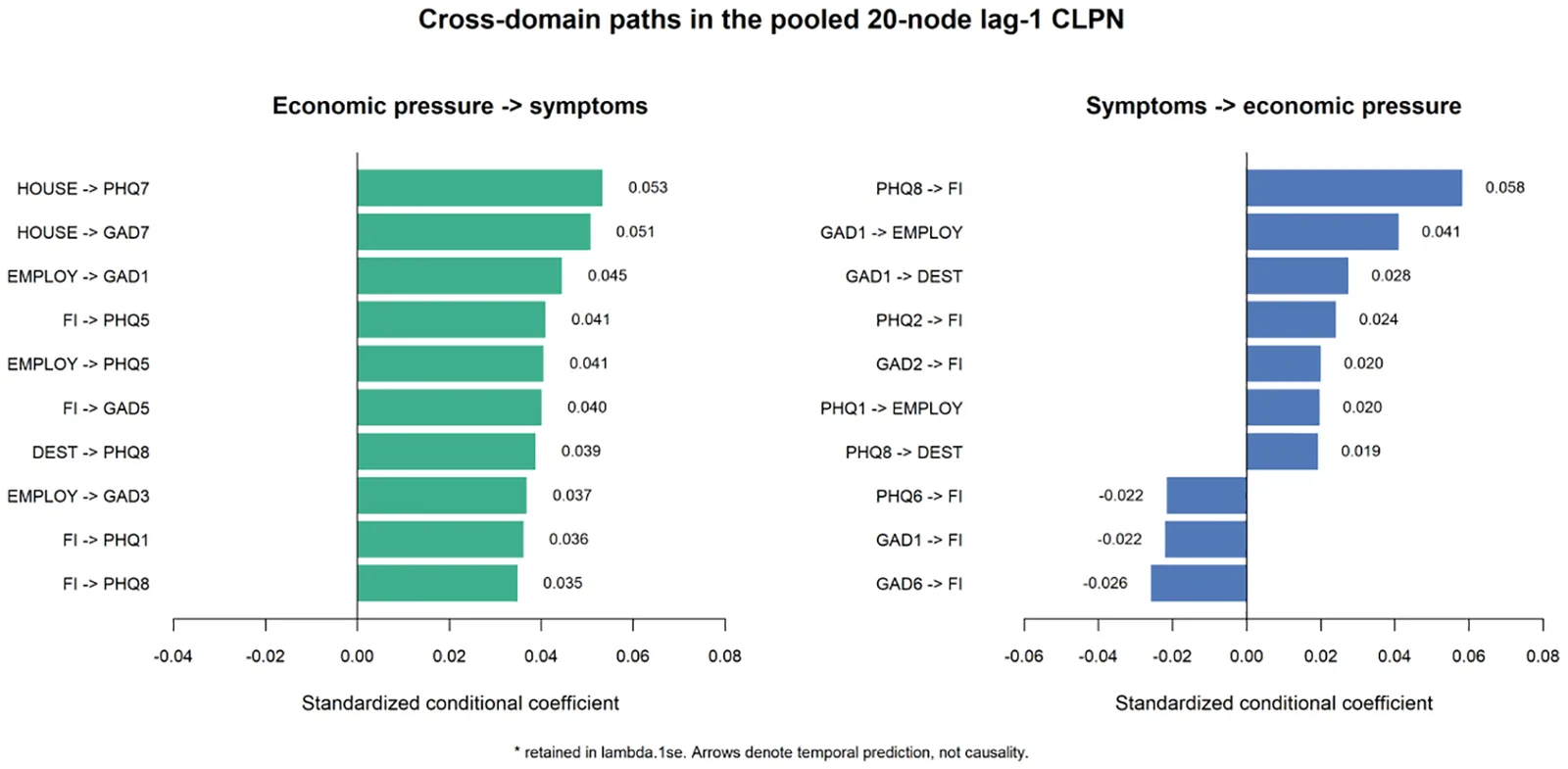
Largest absolute cross-domain paths in the pooled 20-node lag-1 CLPN. Both directions are displayed because the observational CLPN estimates conditional temporal prediction rather than causal direction. Asterisks indicate paths retained in the lambda.1se sensitivity model.

In the direction from symptoms to subsequent economic pressure, larger paths included PHQ8 -> FI (0.058), GAD1 -> EMPLOY (0.041), GAD1 -> DEST (0.028), and PHQ2 -> FI (0.024). However, the weights of these cross-domain paths were clearly lower than those of the major within-symptom and within-economic paths. In the more conservative lambda.1se model, no economic pressure -> symptom paths were retained; only a small number of weak symptom -> economic-pressure paths remained, such as PHQ8 -> FI (0.018) and GAD1 -> EMPLOY (0.013). Therefore, the current results support the conclusion that economic-pressure nodes and the symptom network have limited and weak monthly cross-links, rather than indicating that economic pressure strongly and unidirectionally predicts the symptom network. **Figure 2** displays these cross-domain paths.

### 3.5 Core PHQ/GAD symptom paths

In the extended main network, PHQ/GAD symptoms still showed a clear core temporal predictive structure. Higher-weight symptom paths included PHQ2 -> PHQ1 (0.168), GAD5 -> PHQ8 (0.158), PHQ8 -> GAD5 (0.146), GAD2 -> GAD3 (0.146), GAD3 -> GAD2 (0.140), and PHQ1 -> PHQ2 (0.132). The bidirectional path between PHQ1 and PHQ2 reflects a within-domain loop between anhedonia and depressed mood, whereas the bidirectional path between GAD2 and GAD3 reflects a within-domain loop between inability to control worrying and excessive worry. The largest-weight nonautoregressive cross-lagged paths in the 20-node main network are summarized in **Table 1**.

**Table 1:** Largest-weight nonautoregressive cross-lagged paths in the 20-node main network.

Path	Weight	Relationship type	Retained in lambda.1se
PHQ2 -> PHQ1	0.168	within-depression	Yes
GAD5 -> PHQ8	0.158	anxiety-to-depression	Yes
PHQ8 -> GAD5	0.146	depression-to-anxiety	Yes
GAD2 -> GAD3	0.146	within-anxiety	Yes
GAD3 -> GAD2	0.140	within-anxiety	Yes
HOUSE -> DEST	0.140	within-economic	Yes
PHQ1 -> PHQ2	0.132	within-depression	Yes
PHQ6 -> PHQ2	0.115	within-depression	Yes
GAD1 -> GAD4	0.113	within-anxiety	Yes
GAD7 -> GAD2	0.111	within-anxiety	Yes
PHQ1 -> PHQ7	0.111	within-depression	Yes
DEST -> FI	0.108	within-economic	Yes

**Figure 3:**
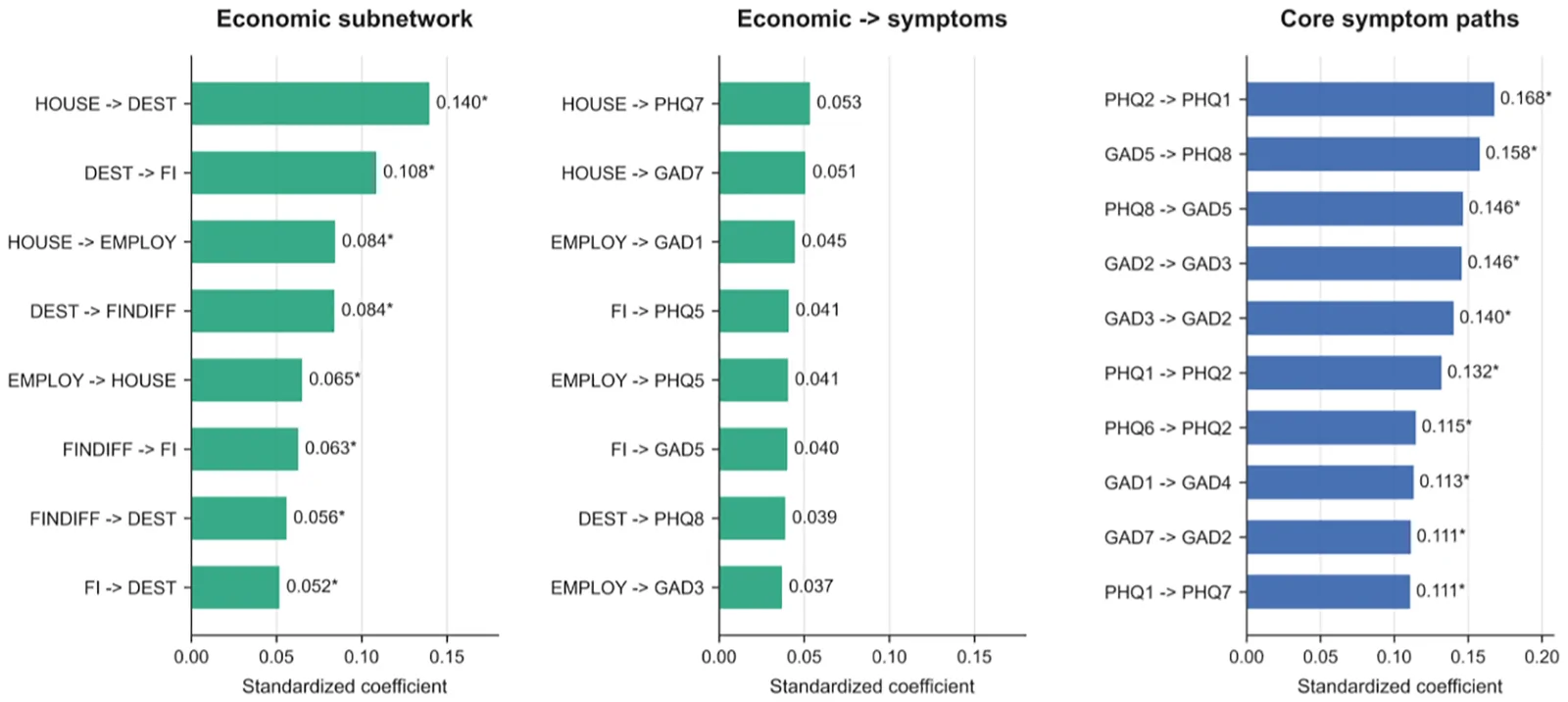
Key paths in the extended network. Asterisks indicate paths retained in the lambda.1se sensitivity model.

Among cross-PHQ/GAD paths, the bidirectional temporal prediction between PHQ8 psychomotor problems and GAD5 restlessness was most prominent. PHQ8 -> GAD5 (0.146) and GAD5 -> PHQ8 (0.158) were both among the stronger paths in the main network and were retained in the lambda.1se sensitivity model, with weights of 0.124 and 0.128, respectively. Therefore, this path can be reported as a candidate transdiagnostic temporal predictive pathway. However, because PHQ8 and GAD5 overlap in motor activation, restlessness, or behavioral regulation, the result should be interpreted as a stable monthly predictive association between content-overlapping symptoms rather than as definitive evidence of an independent transdiagnostic mechanism. The key paths in the extended network are visualized in **Figure 3**.

### 3.6 Centrality and predictability

**Figure 4:**
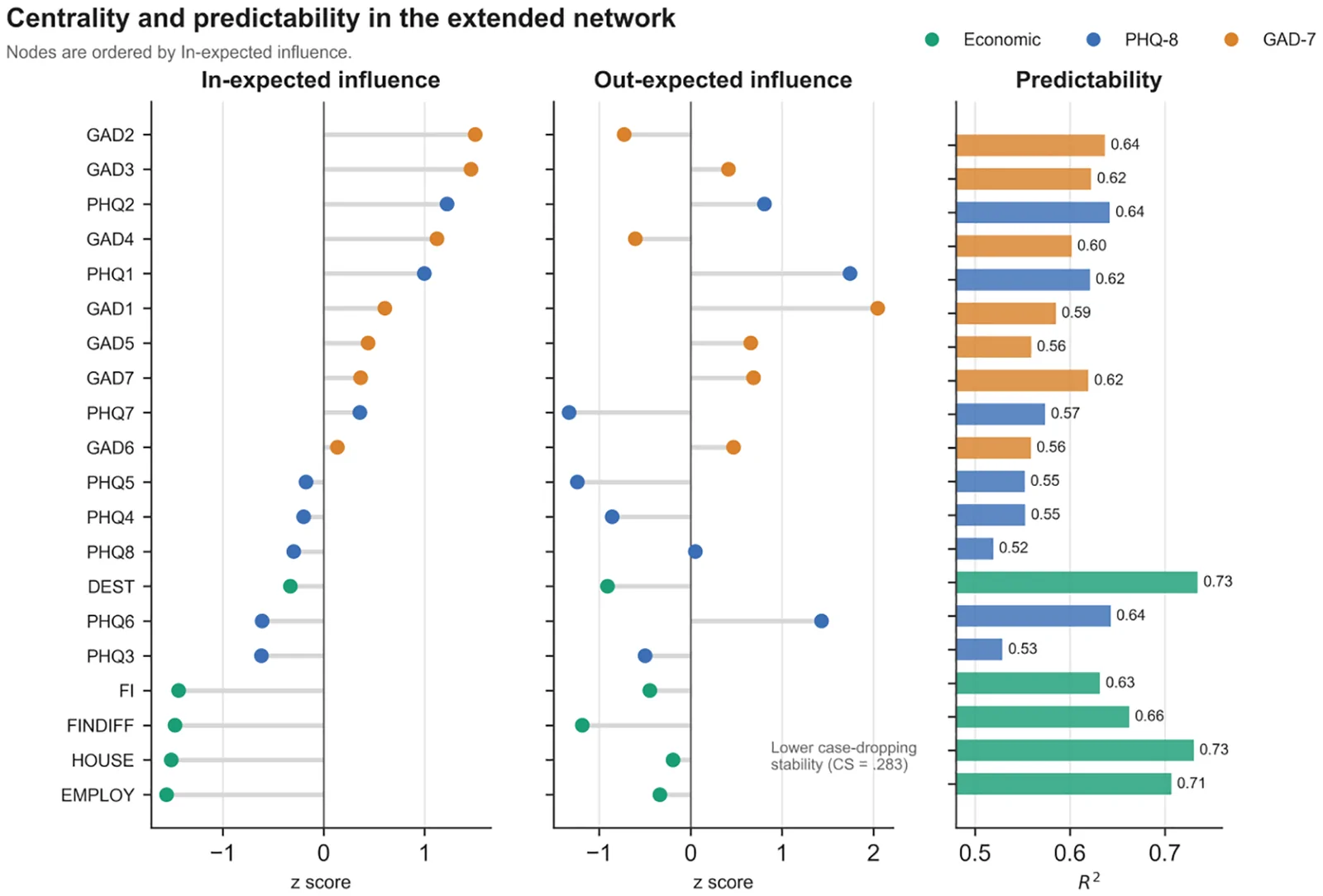
Centrality and predictability of the 20-node main network. The left panel shows In-Expected Influence, the middle panel shows Out-Expected Influence, and the right panel shows node predictability R2. Because the case-dropping stability of Out-Expected Influence was low, this index is interpreted descriptively only.

The In-Expected Influence results showed that nodes with higher incoming expected influence were mainly GAD2 (1.504), GAD3 (1.462), PHQ2 (1.224), GAD4 (1.124), and PHQ1 (0.999). This indicates that, after controlling for other nodes, inability to control worrying, excessive worry, depressed mood, difficulty relaxing, and anhedonia were more likely to serve as input convergence points for subsequent network states. The case-dropping bootstrap indicated good stability of In-Expected Influence (CS = 0.750); therefore, this index was retained as the main descriptive centrality result. This statistical stability does not establish causal importance or treatment relevance. Centrality and predictability results for the 20-node main network are shown in **Figure 4**.

Nodes with higher Out-Expected Influence included GAD1 (2.048), PHQ1 (1.745), PHQ6 (1.433), PHQ2 (0.809), and GAD7 (0.689). However, the stability of Out-Expected Influence was limited (CS = 0.283), exceeding only the minimum acceptable level and not reaching a high stability standard. Therefore, Out-Expected Influence results should be treated as descriptive supplements and should not be used to emphasize certain nodes as stable predictive sources or driving nodes. Nodes with the highest predictability in the extended main network are presented in **Table 2**.

**Table 2:** Nodes with the highest predictability in the extended main network.

Node	R2	Domain
DEST	0.735	economic
HOUSE	0.731	economic
EMPLOY	0.707	economic
FINDIFF	0.662	economic
PHQ6	0.643	depression
PHQ2	0.642	depression
GAD2	0.637	anxiety
FI	0.631	economic

Regarding node predictability, economic-pressure nodes had generally high R2 values, with DEST (0.735), HOUSE (0.731), EMPLOY (0.707), FINDIFF (0.662), and FI (0.631) all showing high predictability. Among symptom nodes, PHQ6 (0.643), PHQ2 (0.642), GAD2 (0.637), GAD3 (0.622), PHQ1 (0.621), and GAD7 (0.619) showed relatively high predictability. These findings suggest that economic-pressure nodes exhibited strong monthly persistence and within-network explainability, whereas some symptoms related to depression, worry, and threat anticipation were also well predicted by other nodes from the previous month.

### 3.7 Robustness and sensitivity analyses

**Figure 5:**
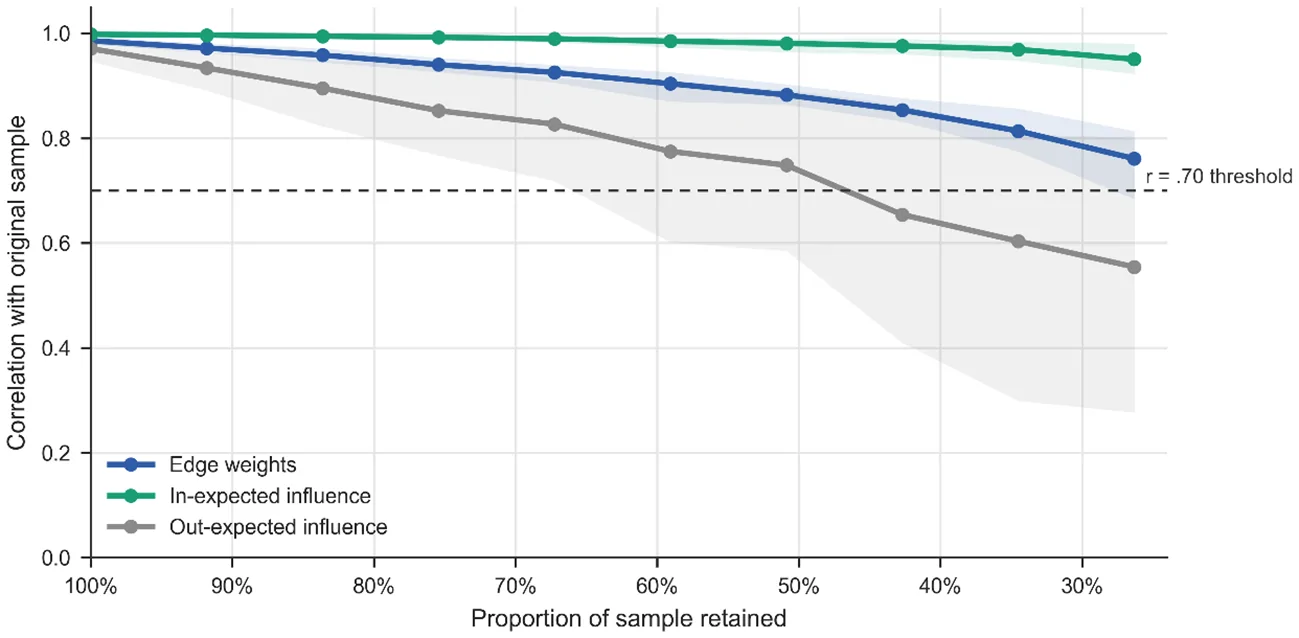
Case-dropping bootstrap stability of the 20-node main network. The dashed line indicates the stability threshold of a correlation of.70. Results from 500 bootstrap samples showed good stability for edge weights and In-Expected Influence, whereas the stability of Out-Expected Influence was relatively limited.

Case-dropping bootstrap analyses showed good edge-weight stability (CS = 0.672), high stability of In-Expected Influence (CS = 0.750), and more limited stability of Out-Expected Influence (CS = 0.361). Accordingly, edge weights and In-Expected Influence were treated as the more reliable network results, whereas Out-Expected Influence was interpreted cautiously as an auxiliary descriptive index. Additional bootstrap difference tests are reported in the **Supplementary Materials**, and case-dropping stability results are shown in **Figure 5**.

The lambda.1se sensitivity analysis yielded a sparser 20-node network with 145 nonzero edges, including 125 nonautoregressive cross-lagged edges, and a mean R2 = .600. The correlation between all cross-lagged edge weights in the lambda.min and lambda.1se models was 0.893, the correlation among the nonzero union of edges was 0.884, and the signed Jaccard index was 0.461. The more conservative model retained core paths such as PHQ2 -> PHQ1, GAD2 -> GAD3, GAD3 -> GAD2, GAD5 -> PHQ8, PHQ1 -> PHQ2, PHQ8 -> GAD5, and HOUSE -> DEST, but weak economic pressure-to-symptom paths were largely not retained. These results indicate that core symptom paths and major within-economic-pressure paths were relatively robust, whereas cross-domain economic pressure-symptom paths were weak and model-dependent. Accordingly, the conservative model defines the interpretation boundary: within-symptom and within-economic-pressure structures are treated as the robust findings, whereas cross-domain paths are treated as exploratory. Network similarity indices from the sensitivity analyses are summarized in **Table 3**.

**Table 3:** Network similarity indices in sensitivity analyses.

Sensitivity model	Correlation of all cross-lagged edges	Correlation of nonzero-union edges	Signed Jaccard
PHQ8 deleted	0.982	0.978	0.948
GAD5 deleted	0.982	0.978	0.923
PHQ8/GAD5 both deleted	0.977	0.971	0.917
20-node lambda.1se	0.893	0.884	0.461

**Figure 6:**
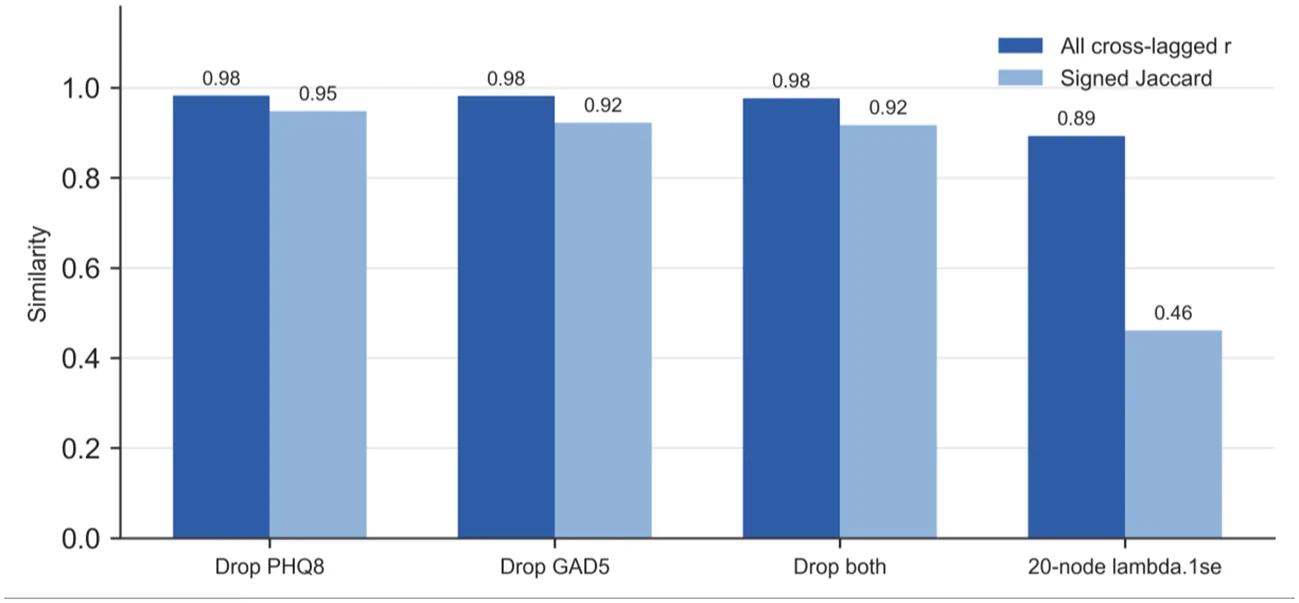
Network similarity in sensitivity analyses.

To address the content similarity between PHQ8 and GAD5 items, additional deletion sensitivity analyses were conducted. Compared with the original 15-node symptom subnetwork, the correlations of all cross-lagged edge weights after deleting PHQ8, deleting GAD5, and deleting both nodes were 0.982, 0.982, and 0.977, respectively, and the corresponding signed Jaccard indices were 0.948, 0.923, and 0.917. These results indicate that the overall symptom-subnetwork structure did not depend on the PHQ8/GAD5 node pair. After both nodes were deleted, more prominent transdiagnostic paths shifted to PHQ6 -> GAD7, GAD6 -> PHQ3, GAD7 -> PHQ6, PHQ6 -> GAD1, and PHQ6 -> GAD2. Overall, the bidirectional PHQ8-GAD5 path was a stable strong path in the main model, but its interpretation should consider item-content similarity; the deletion sensitivity analyses support the robustness of the overall symptom-network structure. Network similarity results from these sensitivity analyses are visualized in **Figure 6**.

## 4 Discussion

### 4.1 Summary of main findings

Using 12 monthly waves from the Changing Cost of Living Study, this study examined temporal predictive associations among cost-of-living pressure, PHQ-8 depressive symptoms, and GAD-7 anxiety symptoms in a pooled lag-1 CLPN. The contribution of this study is to characterize the group-level temporal structure linking multidimensional cost-of-living pressures and depressive-anxiety symptoms, rather than to demonstrate a strong unidirectional effect of economic pressure on mental health symptoms. Overall, the network was characterized mainly by positive temporal predictions, but the strongest and most stable paths were concentrated within economic-pressure nodes and within PHQ/GAD symptoms. Cross-domain economic pressure-symptom paths were generally weak and were not retained under the more conservative lambda.1se specification.

### 4.2 Depressive-anxiety symptom dynamics in the context of cost-of-living pressure

#### 4.2.1 Within-symptom loops as the main dynamic structure

The most notable finding was that, in the pooled network, within-PHQ/GAD temporal predictive paths were stronger than cross-domain paths between economic pressure and symptoms. The reciprocal group-level associations between PHQ1 and PHQ2, and between GAD2 and GAD3, were among the stronger symptom paths; they should not be interpreted as within-person causal loops. This is consistent with network theory, which views depression and anxiety as dynamic interactions among specific symptoms rather than only as latent disease entities (17). In the chronic stress context of a cost-of-living crisis, repeated exposure to uncertainty, perceived resource loss, and threat anticipation may be associated with symptoms such as worry, depressed mood, and anhedonia.

This symptom-dominant pattern also aligns with recent CLPN studies showing that longitudinal links between depression and anxiety are often concentrated in a small set of key symptoms rather than evenly distributed across all items (22–24). The GAD2-GAD3 and PHQ1-PHQ2 pairs were among the stronger reciprocal group-level temporal predictive associations. These findings underscore the value of symptom-level analysis: under similar economic pressure, psychological trajectories may differ according to patterns of symptom co-occurrence and temporal association. Thus, mental health risks during the cost-of-living crisis should be understood not only through PHQ-8 or GAD-7 total scores, but also through dynamic links among symptoms such as worry, anhedonia, and depressed mood.

#### 4.2.2 Interpretation of transdiagnostic symptom channels

Across PHQ/GAD domains, the bidirectional path between PHQ8 and GAD5 was the most prominent and was retained in the conservative lambda.1se model, suggesting a stable monthly link between psychomotor problems and restlessness. However, this path should be interpreted cautiously because both items involve motor activation, restlessness, or behavioral regulation, and may partly reflect item-content overlap rather than an independent transdiagnostic mechanism. Deletion analyses showed that removing PHQ8, GAD5, or both did not substantially change the overall symptom-network structure, indicating that the network did not depend on this item pair.

After both nodes were removed, transdiagnostic paths shifted toward PHQ6, GAD7, GAD1, and GAD2, suggesting that fatigue, threat anticipation, nervousness/anxiety, and worry may also bridge depression and anxiety. This supports a transdiagnostic interpretation in which depression-anxiety comorbidity may be reflected in shared or temporally associated symptom processes rather than diagnostic categories alone (17, 25).

#### 4.2.3 Input-convergence role of worry and core depressive symptoms

GAD2, GAD3, PHQ2, GAD4, and PHQ1 showed relatively high In-Expected Influence, and this index demonstrated good stability. These findings indicate that inability to control worrying, excessive worry, depressed mood, difficulty relaxing, and anhedonia were more likely to serve as input convergence points for subsequent network states. In other words, these symptoms are not necessarily the most stable driving sources, but they are more likely to be jointly predicted by other symptoms and pressure nodes from the previous month. These indices identify statistical positions within the present pooled network, not causal leverage points or evidence of treatment efficacy. Accordingly, symptoms with higher and stable In-Expected Influence should be regarded only as potential foci for future hypothesis-driven research; experimental and external validation are required before any clinical implication is made. The stability of Out-Expected Influence was limited; therefore, nodes such as GAD1, PHQ1, PHQ6, PHQ2, or GAD7 should not be interpreted as stable predictive sources, intervention targets, or clinical priorities. This cautious interpretation is consistent with methodological recommendations in psychological network analysis, which require adequate stability, clear model assumptions, and external validation before any clinical translation (21).

#### 4.2.4 Economic pressure as a contextual social stressor

Limited cross-domain temporal predictive associations were observed between economic-pressure nodes and PHQ/GAD symptoms. At the group level, HOUSE, EMPLOY, and FI were associated with some subsequent symptoms, and symptoms were associated with some subsequent economic-pressure indicators; these paths were generally weak and less stable in the conservative model. Thus, the pooled network did not show a strong or consistent unidirectional temporal predictive association from economic pressure to symptoms. This is consistent with evidence linking economic hardship to mental health through stress load, reduced cognitive bandwidth, constrained choices, and lower social participation, with associations varying by measurement interval, pressure dimension, and model specification (2, 5). Within-economic-pressure paths showed high stability and diffusion among HOUSE, EMPLOY, FI, FINDIFF, and DEST, suggesting an interconnected stress context involving housing risk, poverty/deprivation risk, food insecurity, employment risk, and financial difficulty. This aligns with public health perspectives that food, housing, energy, and employment pressures are jointly relevant to mental health risk (1), and with evidence linking food insecurity, housing insecurity, and financial hardship to poorer mental health (3, 7, 9). Overall, economic pressure may be associated with symptoms such as worry, depressed mood, and anhedonia within a context of persistent threat.

### 4.3 Limitations and future directions

This study has several limitations. Although complete-case analysis may be vulnerable to selection effects if missingness or attrition is systematically related to economic hardship or mental health, we did not formally model the missing-data mechanism or apply imputation. Residual selection effects therefore cannot be excluded. The pooled lag-1 group-average CLPN should be interpreted as group-level monthly predictive patterns rather than within-person causal processes. CLPN paths indicate conditional group-level prediction, not causality or a common individual-level developmental pattern, and may be affected by unmeasured factors such as prior mental health, social support, debt, welfare access, or life events. Repeated lag-1 records from the same participants are also not statistically independent; LASSO regularization and bootstrap assessment provide internal stability evidence but do not fully resolve this dependence. Measurement limitations also remain, including possible PHQ8-GAD5 content overlap and incomplete coverage of economic pressures such as debt, energy poverty, and regional living costs. Educational attainment and citizenship/immigration status were unavailable, so residual confounding by these characteristics cannot be excluded. Although country was included as a covariate, the present pooled analysis was not designed to estimate country-specific networks; pooled results therefore cannot determine whether cross-domain paths differ between France and the United Kingdom.

The descriptive comparison identified country and gender-composition differences, but it was based on only 14 participants without a complete lag-1 record and cannot identify the missing-data mechanism or establish that missingness was ignorable.

Future studies may examine missing-data and attrition patterns and assess appropriate longitudinal or multilevel multiple-imputation strategies. Future studies should use larger and more diverse samples, designs that better distinguish within-person dynamics, and broader socioeconomic and psychological measures. Overall, these findings highlight the need to consider both structural economic pressures and depressive-anxiety symptom dynamics.

## 5 Conclusions

Using a pooled lag-1 CLPN across 12 monthly waves, this study characterized the group-level temporal structure of conditional associations among multidimensional cost-of-living pressures, PHQ-8 depressive symptoms, and GAD-7 anxiety symptoms. Within-economic-pressure persistence and within depressive-anxiety symptom paths were stronger than cross-domain associations. The more stable symptom paths involved anhedonia, depressed mood, uncontrollable worry, and excessive worry, while GAD2, GAD3, PHQ2, GAD4, and PHQ1 emerged as input-convergence points. These findings may inform hypotheses and priorities for future prevention and intervention research. However, this observational secondary analysis cannot establish causal mechanisms or determine the effectiveness of policy or clinical interventions.

## Data Availability

Publicly available datasets were analyzed in this study. The datasets analyzed during the current study are publicly available on the Open Science Framework (OSF) in the Changing Cost of Living Study repository: https://osf.io/d9qb6/. The processed dataset used for analysis is available at https://osf.io/download/3vcnd/.
